# Randomised Controlled Trial of Elective Induction of Labour at 40 and 41 Weeks to Prevent Prolonged Pregnancy

**DOI:** 10.34763/jmotherandchild.20263001.d-25-00024

**Published:** 2026-04-30

**Authors:** Oluwadare Martins Ipinnimo, Olusola Peter Aduloju, Pius Idowu Ade-Ojo, Tope Michael Ipinnimo, Adeyemi Sunday Adefisan, Oluseyi Igbekele Ipinnimo, Babatunde Ajayi Olofinbiyi, Oluwamuyiwa Temitope Fagbohun, Benedict Tolulope Adeyanju, Adewale Temitope Adeyiolu

**Affiliations:** Department of Obstetrics and Gynaecology, Ekiti State University Teaching Hospital, Ado-Ekiti, Nigeria; Department of Community Medicine, Federal Teaching Hospital, Ido-Ekiti, Nigeria; Victoria Hospital, Kirkcaldy, NHS Fife, UK; Department of Haematology, Ekiti State University, Ado-Ekiti, Nigeria; Reddington Multi-Specialist Hospital, Victoria Island, Lagos, Nigeria; Department of Obstetrics and Gynaecology, Afe Babalola University, Ado-Ekiti, Nigeria

**Keywords:** Caesarean section rate, elective, induction of labour, prolonged pregnancy, maternal outcome, foetal outcome

## Abstract

**Background:**

There have been discussions as to the time of elective induction of labour to curb the continuation of pregnancy that might endanger the lives of both the mother and child. This research was conducted to assess foetal and maternal consequences of planned delivery at 40 and 41weeks in women with low-risk singleton pregnancy.

**Material and methods:**

A randomised controlled trial with equal allocation of participants (96 pregnant women in each arm) into 40weeks and 41weeks. Participants were randomised at the antenatal clinic at 39 weeks for induction of labour. The main outcome was the caesarean section rate. Secondary outcomes were maternal (genital tract laceration rate) and foetal (rates of meconium staining of amniotic fluid, SCBU admission, perinatal mortality, birth trauma, birth weight, and neonatal APGAR score at 1 and 5 minutes). Student t-test and chi-square test were used for inter-group comparison.

**Results:**

Incidence of caesarean delivery (26.6% vs. 21.3%; p=0.406), and genital laceration (2.1% vs. 5.6%; p=0.268) did not differ between groups. Significantly higher birth weight was noted among women induced at 41weeks (3.41 ± 0.37kg) than 40weeks (3.28 ± 0.46kg) (p=0.043). Also, there was significant variation in meconium staining of amniotic fluid between 40weeks (11.7%) and 41weeks (25.8%) (p=0.014). Other foetal outcomes showed no significant difference.

**Conclusion:**

Inducing labour at 40weeks is safe for low-risk women as it does not significantly increase the cesarean delivery rate and adverse perinatal outcomes. Therefore, elective induction of labour at 40weeks should be recommended and introduced into obstetric practice without the fear of adverse outcomes.

## Introduction

Elective induction of labour to forestall pregnancy prolongation has been an issue of debate for several years now. The World Health Organisation (WHO) guidelines for the induction of labour are to be followed when there is a medical indication or when the benefits outweigh the potential risks [1,2]. A newborn delivered before 37 completed weeks of gestation is classified as preterm. However, optimal neonatal outcomes are observed among infants born between 39 weeks and 40 weeks plus 6 days of gestation, a period often referred to as full term [3]. The prevalence of adverse neonatal outcomes increases when delivery occurs between 37–38 weeks, and some research suggests the risk of neonatal outcomes rises again beyond 41 weeks of gestation [4]. Obstetricians have reacted in so many ways to the perceived notion that perinatal morbidity or mortality is increasing with the prolongation of pregnancy. The current options of management entail induction at term to avoid pregnancies reaching 42 weeks, routine induction at 42 weeks or before, and selective induction among pregnancies that tests show will have adverse outcomes at 42 weeks. However, it is essential to highlight that the efficacy and risks of some of these approaches have not yet been well studied in controlled trials.

Middleton et al. [5], in their meta-analysis, found that induction of labour at 41 completed weeks may significantly reduce the risk of perinatal and neonatal morbidity and mortality without increasing the caesarean section rate. However, the meta-analysis included few women for perinatal mortality assessment. Also, it included studies conducted in the 1970s, before the introduction of ultrasound dating. A population-based retrospective cohort study done among over 1.2 million pregnant women from Scotland (categorised based on the timing of induction of labour for non-medical indications into 37, 38, 39, 40 and 41 weeks of gestation) showed that induction at these various gestations was associated with reduced perinatal mortality, without an increased risk of caesarean section rate [6].

In contrast, Middleton et al. [5] study shows that induction at or beyond 37 weeks significantly reduces perinatal deaths and stillbirths, lowers caesarean rates, and decreases NICU admissions, while having minimal effect on operative vaginal births, perineal trauma, postpartum haemorrhage, or breastfeeding. It also assessed broader gestational ages (≥37 weeks) rather than focusing solely on 41 weeks. Nonetheless, the American College of Obstetricians and Gynaecologists and the Society for Maternal-Foetal Medicine have recommended against induction of labour for non-medical indications before 39 weeks, as this may further increase perinatal mortality and morbidity [7].

A systematic review and meta-analysis of randomised controlled trials of labour induction at full term (39 weeks to 40 weeks 6 days) in uncomplicated singleton gestations showed that elective labour induction was associated with maternal and foetal benefit compared with expectant management8. Some of the maternal and foetal benefits identified in this review included reduced caesarean section rate, less blood loss and minimal risk of meconium-stained amniotic fluid. Meconium-stained amniotic fluid has been associated with meconium aspiration syndrome, cerebral palsy, seizure, and pulmonary disease [8].

Furthermore, studies have advocated induction of uncomplicated singleton gestations at full term (39 weeks + 0 days to 40 weeks + 6 days), as this will further reduce the burden of perinatal and maternal morbidity and mortality without increasing the caesarean section rate [8,9]. A study done on nulliparous, singleton women with unfavourable cervix by Grobman et al. in 2015 revealed no statistically significant difference in caesarean section rates between elective induction at 39 weeks of gestation compared to expectant management arm using a standardised induction protocol [10]. Induction of labour aims to achieve vaginal delivery, reduce caesarean section and minimise perinatal and maternal morbidity and mortality. There are fewer studies justifying elective induction at 40 weeks 6 days. In contrast, most current literature favours elective induction at 41 weeks of gestation.

The ARRIVE trial has been influential, showing that elective induction at 39 weeks reduced caesarean delivery rates and maternal adverse outcomes in low-risk nulliparous women [11]. Nonetheless, this trial was conducted in a high-resource setting. Its findings may not generalise well to low-and middle-income countries (LMICs), where the burden of maternal and perinatal mortality is higher, and the healthcare systems are significantly different. Despite the proven complexities of post-term pregnancy in settings like Nigeria, where delays to emergency obstetric care access are the norm, there is no context-specific, high-quality evidence to guide elective IOL at or before 41 weeks [10]. There is also a need to figure out whether earlier induction would reduce some of the pitfalls of resource constraints and improve maternal-neonatal outcomes in such settings. Although current international recommendations are founded on robust evidence from HICs, they do not necessarily reflect the reality of care provision in LMICs. Without sophisticatedly designed randomised controlled trials that hold IOL at 40 weeks 6 days and IOL at 41 weeks head-to-head, clinicians and policymakers cannot definitively prove the safest and best approach to the management of low-risk term pregnancies. Therefore, this study aimed to determine whether elective induction at 40 weeks 0 days would provide better maternal and neonatal outcomes than elective induction at 41 weeks 0 days in a healthy singleton woman.

## Material and methods

**Figure 1.**
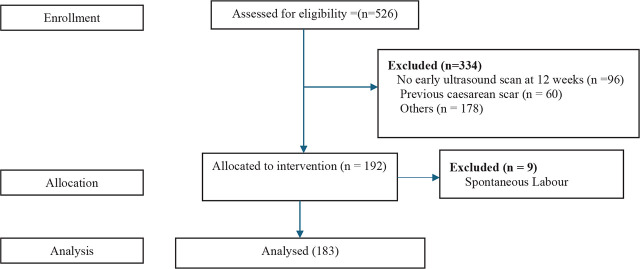
CONSORT Flow Diagram.

This study adheres to the CONSORT guidelines of reporting randomised controlled trials. The CONSORT Flow Diagram (Figure 1) illustrates the details of enrollment, allocation, and analysis.

### Trial design and setting

This is a parallel randomised controlled trial with equal allocation to control and intervention groups. The trial was carried out between August 2023 and February 2024 in the Department of Obstetrics and Gynaecology of Ekiti State University Teaching Hospital, Ado-Ekiti (EKSUTH) and Federal Teaching Hospital, Ido-Ekiti (FETHI) to assess the maternal and perinatal outcomes of women who had elective induction at 40 weeks 0 days and 41 weeks of gestation.

### Participants

The study population included pregnant women attending antenatal clinics in EKSUTH and FETHI. Pregnant women with live singleton fetuses in a cephalic presentation at the gestational age of 40 weeks 0 days to 41 weeks and 3 days gestation from the first day of the last menstrual period and ultrasound biometry before the 12th week of gestation, as well as those with parity of ≤ 4, were included in the study. The study excluded those with previous caesarean section scar, medical conditions such as hypertension, diabetes mellitus and sickle cell anaemia, pregnancies with fetuses having ultrasound-diagnosed congenital abnormalities, and women who had no early ultrasound scan report before 12th weeks.

### Sample size determination

The sample size for this study was determined using the formula for a comparative research study [11]. Based on a failed induction rate of a previous study done in EKSUTH [12], the proportion of participants in the 40 weeks 0 day arm that is expected to exhibit the outcome of interest (20% more than the previous study), the statistical power of 80%, a significance level of 5%, attrition rate of 10%, an estimate of 96 pregnant women would be necessary for each arm of the study. Therefore, a total of 192 subjects who met the inclusion criteria were recruited for the study.

### Patient Allocation/Randomisation

The study ensured that the pregnant women selected met the inclusion criteria and were chosen at 39 weeks and randomised into two (2) groups encompassing 40 weeks 0 day and 41 weeks as Group A and B, respectively, using a blocked randomised number table prepared from computer-generated random numbers kept in sealed envelopes. All these numbered envelopes were placed in a box in serial order. The selected participants were issued a consent form and assigned a sequential number. Patient allocation started with the first sealed opaque envelope and continued sequentially until the last pack was completed. Using this method, equal numbers of participants were assigned to each study arm. The primary investigator conducted this process to maintain the sequence of the envelopes, but the investigator was not directly involved in patient care.

### Intervention

Participants in group A (40 weeks 0-day arm) were admitted at 40 weeks 0 days, while those in group B (41 weeks arm) were admitted at 41 weeks. Participants assigned to each group had an induction of labour done within 24 hours of ward admission after a non-stress test. Cervical assessment was done for all patients on admission.

Only misoprostol was used in the study and administered for cervical ripening when the Bishop score was less than 6. The women in each group received, every six hours, 25 μg of misoprostol (Cytotec; Pfizer Pharm Limited) into the posterior fornix of the vagina until the cervix had a Bishop’s score of 6 or more. This was done to reduce misoprostol’s side effects by using the maximum dose of 75 μg, which is 3 doses. They were asked to inform the nursing staff of any perceived labour pains.

Similarly, Amniotomy was done when the cervix was at least dilated to 4cm, and the presenting part had descended to the cervix. This was done 6 hours after misoprostol administration in subjects with a Bishop score of 6 or higher who had not yet manifested active phase labour. Oxytocin was administered by gravity method at a rate of 2 and 4 mIU/min increments by 2 and 4 mIU/min at 30-minute intervals in multiparas and nulliparous groups until adequate uterine contractions were established. The rate of oxytocin administration was gradually increased to the maximum dose of 32 mIU/minute.

Foetal heart rate was continuously monitored using a cardiotocograph. Labour was actively managed, and recourse to caesarean section was based on standard indications.

### Study Procedure

Medical doctors and midwives in the Department of Obstetrics and Gynaecology of both hospitals were informed and trained on the conduct of the study. All pregnant women were informed about the survey during antenatal clinic visits. Demographic (maternal age, marital status), obstetric (parity), and other data were collected using a pro forma designed by the researchers.

### Outcome Measures

The primary outcome measure was the caesarean section rate following induction of labour. In contrast, secondary outcome measures were other maternal and foetal outcomes. The other maternal outcomes included genital tract laceration rate (vaginal, perineal, and cervical) and the foetal outcomes included rate of meconium staining of amniotic fluid, neonatal intensive care unit admission, perinatal mortality, and birth trauma. Birth weight and neonatal APGAR scores at 1 and 5 minutes were also assessed. Genital tract laceration included all degrees of injury (first to fourth degree), involving vaginal, perineal, and cervical tears. The overall 3.8% rate observed in this study may reflect lower recognition or reporting thresholds, or better perineal support techniques during delivery, compared to other high-resource settings, such as Australia.

### Data Analysis

The data were coded and entered into IBM SPSS Statistics for Windows, Version 21.0. Armonk, NY: IBM Corp. Statistical analyses were conducted. Continuous variables were presented as mean ± standard deviation, while categorical data were presented as frequency and percentages. A comparison between group A (40 weeks) and B (41 weeks) was done using the Student’s t-test for continuous variables. At the same time, the chi-square test or Fisher’s exact test (when more than 20% of the expected counts were less than 5) was used for categorical variables. Results were considered statistically significant when the p-value was less than 0.05.

### Ethical Consideration

Informed consent was obtained from all participants before they completed the study questionnaires. They were assured that, whether they agreed to take part in the research or not, their management would not be compromised. They were also told that they were free to refuse or withdraw from the study at any time without compromising the standard of their treatment. Ethical clearance obtained from the Ethics and Research Committee of EKSUTH (EKSUTH/A67/2017/08/009) and FETHI (ERC/2017/07/24/65B).

### Trial registration

This study had been registered at the Pan African Clinical Trials Registry with registration number: PACTR202306581858623; date of first registration: 23/6/2023; registration link: https://pactr.samrc.ac.za/Researcher/ManageTrials_v2.aspx#:~:text=PACTR202306581858623

## Results

**Figure 2.**
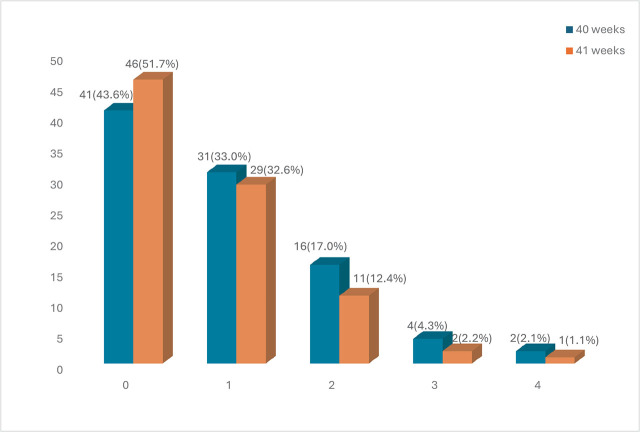
Parity of the study participants.

The total number of participants recruited for this study (from August 2023 to February 2024) was 192, with 96 per arm. Two fell into spontaneous labour at 40 weeks of gestation before the induction day, while seven fell into spontaneous labour at 41 weeks of gestation before the induction of labour day. The mean age ± standard deviation of participants in the 40-week arm was 29.55 ± 3.36 years, while those in the 41-week arm were 29.57 ± 3.79 years (t = −0.038, p-value = 0.970). Figure 2 showed that primigravida was the commonest parity in both groups, accounting for 41 (43.6%) in the 40-week group and 49 (51.7%) in the 41-week group (χ^2^ = 2.145, *p*-value = 0.709). The baseline distribution of participants did not differ significantly across groups. Marital status, religion, ethnicity, level of education, occupation, and booking status did not vary between groups induced at 40 weeks and 41 weeks of gestational age (p-value > 0.05).

Table 1 showed that a percentage of women induced at 40 weeks had caesarean delivery (26.6%) compared to those induced at 41 weeks (21.3%). However, this difference was not statistically significant (p-value = 0.406). In this study, about one-fourth (24.0%) of 44 participants had a caesarean delivery.

**Table 1.** Mode of delivery among participants who had induction of labour at 40 and 41 weeks.

**Mode of delivery**	**Gestational Age**		**Total**	**χ^2^**	***p* value**
	
	**40 weeks n=94 (%)**	**41 weeks n=89 (%)**	**N (%)**		
Vaginal delivery	69 (73.4)	70 (78.7)	139 (76.0)	0.689	0.406
Caesarean section	25 (26.6)	19 (21.3)	44 (24.0)		

χ^2^: Chi-square test.

Table 2 summarises the genital tract laceration rate concerning the timing of IOL. A higher percentage of 5 (5.6%) of women induced at 41 weeks had genital lacerations than those 2 (2.1%) at 40 weeks, but this was not statistically significant (*p*-value = 0.268).

**Table 2.** Genital tract laceration among participants who had induction of labour at 40 and 41 weeks.

**Genital tract laceration**	**Gestational Age**		**Total**	**χ^2^**	***p* value**
	
	**40 weeks n = 94(%)**	**41 weeks n = 89 (%)**	**N (%)**		
**Vaginal laceration**					
No	94 (100.0)	89 (100.0)	183 (100.0)		
**Cervical laceration**					
Yes	1 (1.1)	3 (3.4)	4 (2.2)	1.138^f^	0.358
No	93 (98.9)	86 (96.6)	179 (97.8)		
**Perineal laceration**					
Yes	2 (2.1)	3 (3.4)	5 (2.7)	0.266^f^	0.676
No	92 (97.9)	86 (96.6)	178 (97.3)		
Genital laceration^*^					
Yes	2 (2.1)	5 (5.6)	7 (3.8)	1.514^f^	0.268
No	92 (97.9)	84 (94.4)	176 (96.2)		

χ^2^: Chi square test;

f: Fisher’s exact test;

* Some participants had both cervical and perineal lacerations.

Table 3 shows the relationship between the timing of induction and neonatal APGAR score at one and five minutes. A lower percentage of participants in the 40-week arm 3 (3.2%) had neonates with an APGAR score at 5 minutes <7, compared with 6 (6.7%) in the 41+-week arm, but this difference did not reach statistical significance (*p*-value = 0.320). Similarly, the APGAR score at 1 minute did not differ in the two groups (*p*-value = 0.295).

**Table 3.** Apgar scores of neonates among mothers who had induction of labour at 40 and 41 weeks.

**APGAR score**	**Gestational Age**		**Total**	**χ^2^**	***p* value**
	
	**40 weeks n = 94(%)**	**41 weeks n = 89 (%)**	**N (%)**		
**At 1 minute**					
< 7	9 (9.6)	13 (14.6)	22 (12.0)	1.095	0.295
≥ 7	85 (90.4)	76 (85.4)	161 (88.0)		
Mean ± SD	7.55 ± 1.03	7.30 ± 0.98		1.675^t^	0.096
**At 5 minutes**					
< 7	3 (3.2)	6 (6.7)	9 (4.9)	1.232^F^	0.320
≥ 7	91 (96.8)	83 (93.3)	174 (95.1)		
Mean ± SD	9.48 ± 1.06	9.18 ± 1.25		1.753^t^	0.081

χ^2^: Chi square test;

f: Fisher’s exact test;

t: Independent Samples T test.

Table 4 summarises the relationship between IOL timing and other neonatal outcomes. Statistically significantly higher birth weight was noted among women induced at 41 weeks with a mean birth weight (3.41 ± 0.37kg) than at 40 weeks (3.28 ± 0.46kg) (t = −2.037, p-value 0.043). There was also no significant difference noted concerning respiratory distress (40weeks = 14, 14.9%; 41weeks = 15, 16.9%; p-value = 0.840), special care baby unit (SCBU) admission (40weeks = 13, 13.8%; 41weeks = 10, 11.2%; p-value= 0.597), outcome of neonate at discharge (40weeks = 100% alive; 41weeks = 100% alive) and degree birth trauma (40weeks = 3, 3.2%; 41weeks = 2, 2.3%; p-value = 1.000). However, significant variation occurred between women induced at 40 weeks 11(11.7%) and 41 weeks 23(25.8%) in terms of meconium staining of amniotic fluid (X^2^ = 6.043, p-value = 0.014).

**Table 4.** Other neonatal outcomes among the study participants.

**Variable**	**Gestational Age**		**Total**	**χ^2^**	***p* value**
	
	**40 weeks n = 94(%)**	**41 weeks n = 89 (%)**	**N (%)**		
**Birth weight (kg)**					
Mean ± SD	3.28 ± 0.46	3.41 ± 0.37		−2.037^t^	0.043^*^
**Respiratory distress**					
Yes	14 (14.9)	15 (16.9)	29 (15.8)	0.132	0.840
No	80 (85.1)	74 (83.1)	154 (84.2)		
**Meconium staining of amniotic fluid**					
Yes	11 (11.7)	23 (25.8)	34 (18.6)	6.043	0.014
No	83 (88.3)	66 (74.2)	149 (81.4)		
**SCBU admission**					
Yes	13 (13.8)	10 (11.2)	23 (12.6)	0.280	0.597
No	81 (86.2)	79 (88.8)	160 (87.4)		
**Outcome at discharge**					
Alive	94 (100.0)	89 (100.0)	183 (100.0)		
Dead					
**Birth trauma**					
Dislocation	3 (3.2)	2 (2.3)	5 (2.8)	0.134^f^	1.000
None	91 (96.8)	85 (97.7)	176 (97.2)		

χ^2^: Chi square test;

f: Fisher’s exact test;

t: Independent Samples T test;

*: *p* value <0.05.

## Discussion

Currently, the United States of America and many parts of the world are experiencing an increase in elective obstetric interventions before 41 weeks, despite limited research [13,14]. There are a few risks and benefits to evidence-based practice [15]. This prospective randomised controlled study was conducted in Nigeria to determine maternal and perinatal outcomes of elective induction at 40 weeks and 41 weeks of gestation. Parity distribution does not vary significantly between the two groups. This was also similar to a retrospective cohort study by Hong [16]. This is important as induction is likely to be successful in multiparous women compared to nulliparous women due to the favourability of their cervix.

This study found no statistically significant difference in the caesarean section rate at 40 and 41 weeks of gestation. Given the strict method of cervical ripening employed in both arms of the study, this may suggest that labour induction and progress to delivery are not affected by the gestational age of the fetus at delivery. The result was like a systematic review and meta-analysis conducted by Saccone et al. in 2015 [8]. Grobman et al. [10] found that elective induction of labour at 39 weeks in low-risk nulliparous women was associated with a lower rate of caesarean delivery and fewer maternal complications, without increasing neonatal risks.

Furthermore, the result of this study differs from earlier studies by Carlson et al. and Lee et al. in 2021 and 2022, respectively, which found that the caesarean section rate increased with elective induction of labour [17,18]. The reason for the higher caesarean section rate in these previous studies may be related to the comparison groups: women undergoing induction of labour were compared with women who had spontaneous labour. However, the clinical reality is not a choice between induction and spontaneous labour. However, between induction and continuing pregnancy, that is expectant management with the potential for either spontaneous or induced labour and delivery at a later gestational age [16]. Most studies that increased caesarean section rates were observational or retrospective, and their results were based on patient records. The induction protocol during those studies was artificial membrane rupture and oxytocin titration compared to prostaglandins, which are now widely available and are better cervical ripening agents. However, a recent randomised controlled trial by Gobman et al. in 2018 showed a lower caesarean section rate at 39 weeks compared to 41 weeks of gestation [19].

This study showed no significant difference in neonatal APGAR scores for those induced at 40 and 41 weeks of gestation. This finding is similar to that of Grobman et al. [10] in 2019, who compared elective induction at 39 weeks with expectant management. Additionally, genital tract laceration did not show any significant difference between those induced at 40 and 41 weeks. This is like the findings of Hong et al. [16]. However, this contrasted with a study done by El-Sayed et al. [6] in 2020, in which there were decreased odds for severe genital laceration at 40 weeks’ gestational age when compared to expectant management. Additionally, the Swedish nationwide study showed that active induction at 41 weeks significantly reduced perinatal death (0.9/1,000 vs 1.7/1,000; adjusted RR 0.52) and composite adverse neonatal outcomes, while slightly increasing emergency cesarean sections. The difference reflects the larger sample size and population-level assessment in the Swedish study, which enabled the detection of rare but critical outcomes [20]. Although evidence indicates that induction of labour does not elevate the risk of severe perineal lacerations, with similar rates of third- and fourth-degree tears across groups [21]. This challenges assumptions that induction accelerates labour in ways that heighten perineal trauma. The reduced caesarean and macrosomia rates further suggest potential benefits without added perineal harm.

Higher birth weight was noted in this study among women induced at 41 weeks with a mean birth weight (3.41±0.37kg) as compared to 40 weeks (3.28±0.46kg). This finding is similar to that found in the study by Grobman et al. [10]. Birthweight is expected to increase with higher gestational age. There was no statistically significant difference noted concerning respiratory distress, SCBU admission, outcome of the neonate at discharge and degree of birth trauma as compared to results obtained by Hong et al. and Grobman et al. in their studies [10,16]. The ARRIVE study shows that elective induction at 39 weeks did not worsen perinatal outcomes, yet its widespread adoption in the United States has been linked to hospital strain and “negative spillover” effects that indirectly compromise care [22]. This pattern of elective induction is not fully justified in Nigeria, where it is rare and most inductions are medically indicated for cultural reasons. Nevertheless, similar systemic pressures arise through different mechanisms. Nigeria’s maternity units frequently face overcrowding, staffing shortages, and limited monitoring capacity, meaning any increase in induction, whether elective or medically indicated, can exacerbate workload and compromise prompt obstetric care.

More participants in the 41-week Group had meconium-stained amniotic fluid compared to those in the less than 40-week Group. This finding is comparable to a meta-analysis of various randomised controlled induction trials at 40 weeks’ gestational age compared to expectant management done by Saccone et al. [8] in 2015. This finding relates to the earlier gestational age at induction (40 weeks), when the fetus has not yet passed meconium in utero, compared to those at less than 40 weeks. Meconium-stained amniotic fluid has been associated with meconium aspiration syndrome, cerebral palsy, seizures, and pulmonary disease [23]. 5% of infants with meconium staining of amniotic fluid develop meconium aspiration syndrome, and about 4% of the infants with the syndrome die, accounting for 2% of perinatal deaths [8].

The study’s strengths include its prospective, parallel randomised controlled design, reducing selection bias and enabling robust comparison between elective induction at 40 and 41 weeks. The participants and the managing physicians were not blinded as they were aware of the gestational age at induction. A standardised induction protocol using misoprostol, amniotomy, and graded oxytocin ensured consistent intervention, while continuous fetal monitoring via cardiotocography enhanced safety and accuracy of outcomes. Findings offer context-specific evidence from Nigeria, addressing maternal and neonatal outcomes in low- and middle-income settings. Early ultrasound for gestational dating improved accuracy, and recruitment from two tertiary hospitals increased representativeness and generalisability. Collectively, these methodological and contextual strengths support the reliability and applicability of the study’s conclusions. Although this study is methodologically sound, it presents certain limitations. It was conducted in two tertiary hospitals in Ekiti State, limiting generalisability to other settings. Despite randomisation, blinding was not feasible, potentially introducing observer bias. Only misoprostol was used for induction, excluding comparisons with different methods. Excluding high-risk pregnancies also narrows applicability. Moreover, short-term outcomes were assessed without long-term follow-up, and the study may have been underpowered for rare secondary outcomes. Patient satisfaction and cost-effectiveness were not evaluated. These factors limit the broader applicability and comprehensive understanding of elective induction at 40 versus 41 weeks of gestation.

## Conclusion

Induction of labour at 40 weeks in low-risk women did not result in a significant increase in the rate of cesarean delivery and adverse perinatal outcomes. Based on the findings shown in this study, elective induction of labour at 40 weeks should be encouraged in obstetric practice without fear of increasing the caesarean section rate or adverse perinatal outcome. However, the decision to enact this elective induction of labour intervention may be conditioned upon the women’s values, goals, and preferences, the resources available, and the setting in which the intervention will be implemented.
